# In crystallo observation of three metal ion promoted DNA polymerase misincorporation

**DOI:** 10.1038/s41467-022-30005-3

**Published:** 2022-04-29

**Authors:** Caleb Chang, Christie Lee Luo, Yang Gao

**Affiliations:** Department of Biosciences, Rice University, Houston, TX 77005 USA

**Keywords:** X-ray crystallography, DNA synthesis

## Abstract

Error-free replication of DNA is essential for life. Despite the proofreading capability of several polymerases, intrinsic polymerase fidelity is in general much higher than what base-pairing energies can provide. Although researchers have investigated this long-standing question with kinetics, structural determination, and computational simulations, the structural factors that dictate polymerase fidelity are not fully resolved. Time-resolved crystallography has elucidated correct nucleotide incorporation and established a three-metal-ion-dependent catalytic mechanism for polymerases. Using X-ray time-resolved crystallography, we visualize the complete DNA misincorporation process catalyzed by DNA polymerase η. The resulting molecular snapshots suggest primer 3´-OH alignment mediated by A-site metal ion binding is the key step in substrate discrimination. Moreover, we observe that C-site metal ion binding preceded the nucleotidyl transfer reaction and demonstrate that the C-site metal ion is strictly required for misincorporation. Our results highlight the essential but separate roles of the three metal ions in DNA synthesis.

## Abstract (web summary)

By observing DNA polymerase misincorporation with time-resolved crystallography, the authors visualize three-metal ion dependent polymerase catalysis and identify A-site metal-mediated primer alignment as a key step in nucleotide discrimination.

## Introduction

**Fig. 1:**
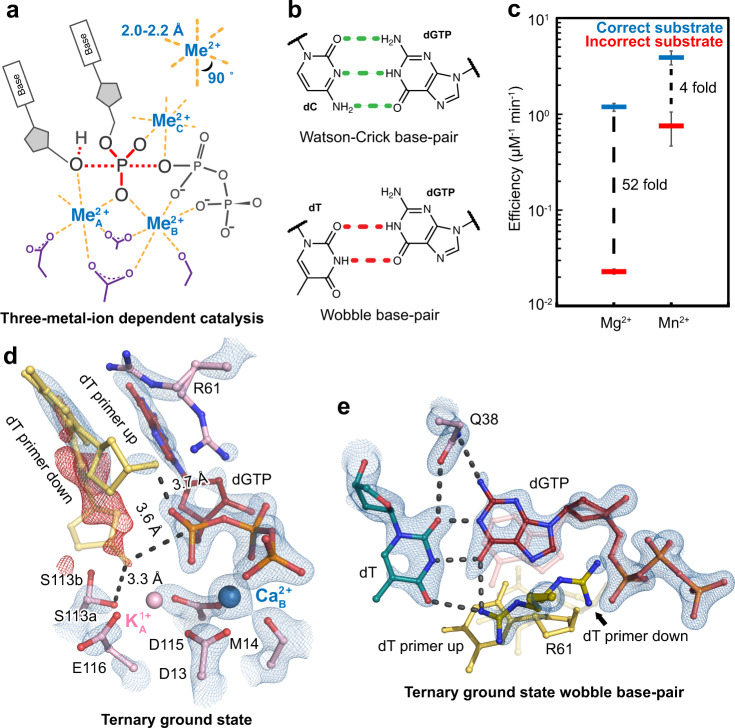
Mechanisms of polymerase η catalysis and misincorporation. **a** Scheme of three-metal-ion dependent enzyme catalysis and transition state stabilization. Divalent metal ions optimally bind to their ligands with bond lengths of 2.0–2.2 Å at 90°. **b** Geometries of dGTP:dC Watson Crick base-pair and dGTP:dT wobble base-pair. **c** DNA polymerase fidelity in the presence of Mg^2+^ versus Mn^2+^ based on steady-state kinetics. The bars represent the mean of duplicate measurements for the catalytic efficiencies (k_cat_/K_M_) for incorporation of dATP (blue) and dGTP (red) opposite dT for Pol η with Mg^2+^ and Mn^2+^(Supplementary Fig. 1). The errors bars represent the standard deviation for the measurements. The distance between the respective catalytic efficiencies is a measure of discrimination. **d** Structure of Pol η ground state complexed with Ca^2+^. The 2F_o_-F_c_ map for the primer up conformation, Me^2+^_A_, Me^2+^_B_, dGTP, and catalytic residues (blue) was contoured at 2 σ (σ values represent r.m.s. density values). The F_o_-F_c_ omit map for the down conformation of the primer (red) was contoured at 3 σ. **e** dGTP:dT wobble base-pairing (red and dark cyan), dATP (pink), and primer (yellow) in the Pol η ground state. The 2F_o_-F_c_ map for dGTP, template dT, two conformations of R61, and Q38 (blue) was contoured at 1.8 σ.

DNA polymerases catalyze the essential biological process of DNA replication. Based on their amino acid sequences, these vital enzymes have been classified into A, B, C, D, X, Y, reverse transcriptase (RT), and primase and polymerase (PrimPol) families^1^. Despite their various biological roles and subunit compositions, all DNA polymerases contain similar active sites and catalyze the metal-ion dependent nucleotidyl transfer reaction via similar kinetic pathways. Moreover, most DNA polymerases resemble a right hand architecture with the active site residing in the palm domain, a thumb domain that binds the primer:template DNA, and a finger domain that interacts with the nascent base-pair. All polymerase active sites contain two to three conserved acidic residues that coordinate two metal ions (Fig. 1a). The A-site metal ion (Me^2+^_A_) bridges the primer 3′-OH and the substrate α-phosphate to promote deprotonation and nucleophilic attack, while the B-site metal ion (Me^2+^_B_) stabilizes the triphosphate motif of the incoming deoxynucleotide triphosphate (dNTP). Drawn from many early crystal structures, it was believed that two metal ions were required and sufficient for catalysis;^2^ however, recent time-resolved crystallography experiments revealed that polymerases acquired a third metal ion (Me^2+^_C_) during catalysis, suggesting a three-metal-ion dependent mechanism^3–6^ (Fig. 1a). The observed Me^2+^_C_ bound between the α- and β-phosphates and may have driven α-β-phosphate bond breakage^3^. Similarly, a two-metal-ion dependent catalysis mechanism has also been proposed for many nucleases; however, additional divalent metal ions were observed in RNaseH^7^ and Endonuclease V^8^ with time-resolved crystallography. In addition, three metal-ion dependent catalysis was also observed in non-nucleic acid enzymes, fructose-1,6-bisphosphatase^9^ and type I terpenoid synthases^10^, suggesting a widespread mechanism in promoting phosphorus-oxygen bond breakage and formation.

During DNA replication, polymerases are faced with the task of incorporating the correct nucleotide from a pool of nearly identical-looking dNTPs dictated by the template bases. Structural studies with various polymerases have suggested that base tautomerization^11,12^, solvation of the active site^13^, and non-Watson-Crick geometries such as Hoogsteen^14^, syn-trans^15^, and wobble base^15,16^ geometries (Fig. 1b) help to stabilize mismatches and reduce steric clashes. Intrinsic fidelity in most polymerases, however, is much higher than what can be provided by the difference in base-pairing energy^1,17,18^. Pre-steady-state kinetics have suggested that a rate-limiting conformational step proceeds catalysis and might be the key in substrate discrimination^19,20^. Based on early structural work, an open-to-closed transition occurs in the finger domain in most replicative polymerases following dNTP binding^21^. It was thus proposed that finger domain closure was the key step during misincorporation^19,20,22^. This finger domain movement, however, is absent in Y-family polymerases and was too fast to be rate-limiting in neither correct nor incorrect nucleotide incorporation^1,23–25^. Subsequently, biophysical studies of X-family DNA polymerase β (Pol β) and Y-family Dpo4 polymerase have suggested that a local “micro” conformational change within the finger domain might play a role in polymerase fidelity^26–28^. On the other hand, work from Tsai, Johnson, Goodman, and co-workers argued that a rate-limiting conformational change was unnecessary to explain the kinetic data and that a kinetic balance between incoming dNTP release and nucleotidyl transfer could account for polymerase fidelity^25,29–31^. Other fluorescent studies of Pol β have suggested that the rate-limiting step requires a functional primer^32^ and have proposed that the rate-limiting step is dependent on primer alignment^33^. Time-resolved crystallography can potentially reveal information about the dynamic catalytic process at atomic resolution^4–7,34–36^. Structural snapshots of Pol β during misincorporation have revealed that an incoming dATP formed a buckle base-pair with a template dG and that the primer terminus and α-phosphate in both the substrate and product states were misaligned^5^. However, intermediate structures right before and right after the chemistry step were not captured and how the substrates were aligned prior to nucleotidyl attack remains elusive. Moreover, in contrast with correct nucleotide incorporation^5,34^, the Me^2+^_C_ was not captured during the misincorporation process.

DNA polymerase η (Pol η) is a Y-family polymerase involved in DNA translesion synthesis against UV-induced lesions^37^. Individuals with mutations on the Pol η gene develop a hypersensitivity to UV-radiation and often have a predisposition for xeroderma pigmentosum and skin cancer^38,39^. In addition, Pol η is involved in antibody generation as well as prevents tumor cellular arrest and confers drug resistance^40–45^. The Pol η time-resolved crystallographic model system has been used extensively to investigate the catalytic mechanism of DNA polymerases^4,34,36,46,47^. Utilizing this Pol η system, the reaction process of correct nucleotide incorporation has been captured and a transiently bound Me^2+^_C_ has been proven essential for catalysis^4,34^. Static structures of Pol η incorporating incorrect nucleotides have also been reported^48^. The incorrect substrate dGMPNPP formed a wobble base with the template dT, which was stabilized by protein sidechains within the Pol η active site. However, the reaction process was not resolved and the key structural determinant for fidelity remained unclear. In this study, we determine 34 atomic-resolution structures from time-resolved X-ray crystallography and elucidate the entire misincorporation process by Pol η with Mg^2+^ and Mn^2+^. Our results suggest that primer 3´-OH-substrate alignment by the Me^2+^_A_ is the key step in substrate discrimination. We also highlight Mn^2+^’s superior ability in aligning the 3′-OH, explaining its error-prone nature compared to Mg^2+^. Furthermore, we show that the Me^2+^_C_ binds before the reaction, and support its essential role in promoting catalysis.

## Results

### Biochemical assays

Pol η has previously been shown to be more error-prone on DNA substrates that contain the WA motif (5′-AA or 5′-TA) on the primer termini^48^. We reasoned that the error-prone WA substrate would be useful in facilitating mechanistic dissections of misincorporation. Steady-state kinetic assays were conducted to confirm Pol η’s discrimination against incorrect substrate insertion on a DNA substrate that contained a WA motif at the primer termini. With Mg^2+^, the steady-state efficiencies indicated that the incorrect substate was discriminated by ~52-fold against the correct substrate, similar to the previous report^48^ (Fig. 1c and Supplementary Fig. 1). It is widely known that Mn^2+^ can support polymerase catalysis and stimulate misincorporation. Therefore, we characterized the efficiency of correct and incorrect substrate insertion by Pol η with Mn^2+^. The kinetic assays indicated that correct nucleotide incorporation efficiency was roughly similar in the presence of Mg^2+^ or Mn^2+^, but Mn^2+^ strongly increased incorrect nucleotide incorporation efficiency. Incorporation of dGTP against dT in the presence of Mn^2+^ was only four-fold less efficient than the incorporation of dATP across dT, resulting in a ~13-fold decrease in discrimination compared to that with Mg^2+^. These results confirmed that Pol η is error-prone and that Mn^2+^ significantly reduces substrate discrimination.

### dGTP binds as a wobble base in Pol η’s active site

Following our previous protocol^49^, we grew Pol η crystals complexed with a DNA substrate that contained a WA motif at the primer termini and a dT as the nascent template, incorrect dGTP substrate, and the inhibitory divalent metal ion, Ca^2+^, at a low pH of 6.0 (Supplementary Fig. 2a). The overall structure including the finger domain looked identical to that of correct nucleotide incorporation, having an r.m.s.d. of 0.2 Å (Supplementary Fig. 3). Within the active site, the incorrect dGTP was assigned with 80% occupancy according to its density. In the ground state, *t* = 0 s, the Me^2+^_A_ site was occupied at 30% by a monovalent K^1+^, and the Me^2+^_B_ site was occupied at 80% by a divalent Ca^2+^ (Fig. 1d). This structure was similar to the ground state during correct nucleotide incorporation (Supplementary Fig. 4a), in which the Me^2+^_A_ site remained partially occupied by K^1+^ while the Me^2+^_B_ site was occupied by Ca^2+^ at 100%. In our mismatch structure, the majority of the R61 side chain was rotated away from the triphosphate group of dGTP, and the primer end existed in two conformations, up and down like in the static structure with a nonhydrolyzable dGMPNPP and two Mg^2+^ ions^48^ (Supplementary Fig. 4c). In the up conformation, the primer 3′-OH was located 3.7 Å above the substrate α-phosphate while the sugar moiety interacted with R61. In the down conformation, the 3′-OH existed 3.6 Å away from the K^1+^ and 3.3 Å away from S113 (Fig. 1d and Supplementary Fig. 4e). Assigning 15% of the 3′-OH in the down conformation eliminated any significant F_o_-F_c_ peaks around the primer terminus. In contrast, during correct nucleotide incorporation, all of the primer was in the down conformation stabilized by S113 (Supplementary Figs. 4a and f). Regarding base-pair geometry of our mismatch structure, the incorrect substrate dGTP and template dT formed a wobble base-pair, in which the base of the incoming dGTP shifted 1.6 Å towards the minor groove while the template dT shifted 0.9 Å towards the major groove, compared to the ground state structure with the correct substrate dATP (Fig. 1e and Supplementary Figs. 4b and d). Hydrogen bonds between dGTP and the protein sidechains Q38 and R61 possibly stabilized the wobble base-pair. Furthermore, due to the wobble base geometry, there was a reduction in pi-stacking interactions between the incoming dGTP and primer aromatic base.

### In crystallo observation of misincorporation by Pol η in the presence of Mg^2+^

**Fig. 2:**
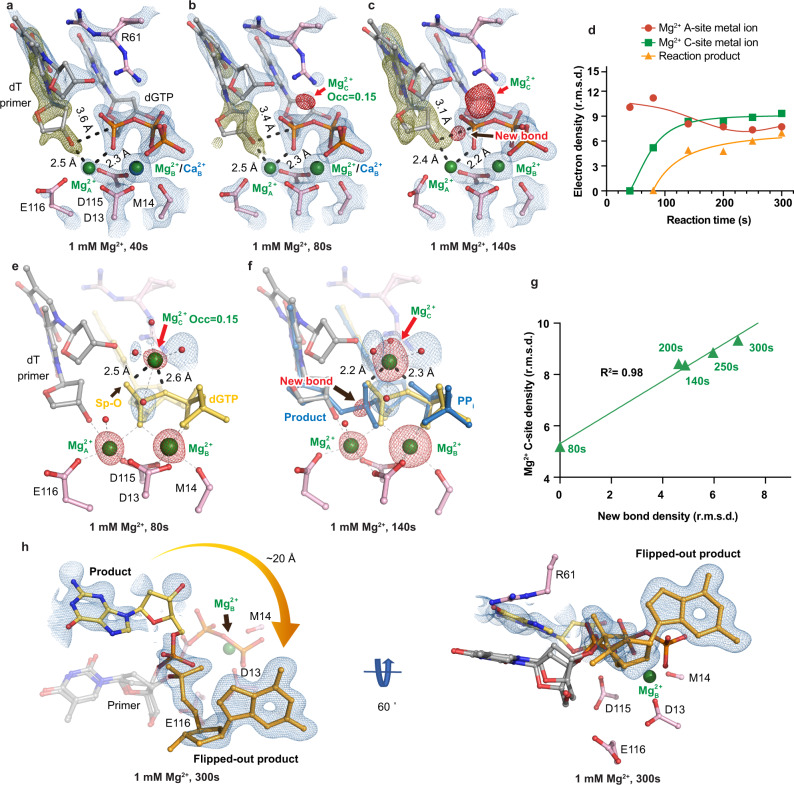
In crystallo visualization of Pol η misincorporation with Mg^2+^. **a**–**c** Structures of Pol η during in crystallo catalysis after 1 mM Mg^2+^ soaking for 40 s (**a**), 80 s (**b**), and 140 s (**c**). The 2F_o_-F_c_ map for the primer up conformation (blue) was contoured at 1.2 σ. The 2F_o_-F_c_ map for the Me^2+^_A_, Me^2+^_B_, dGTP, and catalytic residues (blue) was contoured at 2 σ in (**a**–**c**). The F_o_-F_c_ omit map for the down conformation of the primer (green) was contoured at 3.3 σ in (**a**–**c)**. The F_o_-F_c_ omit map for all the Mg^2+^_C_, and newly formed bond (red) was contoured at 4 σ. **d** Timescale of Mg^2+^_A_, Mg^2+^_C_ binding and reaction product in crystallo with Mg^2+^. **e**, **f** Observation of the Mg^2+^_C_ after 1 mM Mg^2+^ soaking for 80 s (**e**) and 140 s (**f**). **g** Correlation (*R*^2^) between the newly formed bond and the Mg^2+^_C_ binding. **d**, **g** The data points represent singular measurements for r.m.s. density values of electron density. In (**e**, **f**), the 2F_o_-F_c_ map for the water molecules that were coordinated by the Mg^2+^_C_ was contoured at 0.7 σ. The F_o_-F_c_ omit map for the Mg^2+^_C_, new bond, and Mg^2+^_A_ and Mg^2+^_B_ was contoured at 4 σ. **h** Alternative product state of Pol η in crystallo with 1 mM Mg^2+^ at 300 s. The 2F_o_-F_c_ map was contoured at 1 σ.

To start the reaction, we equilibrated Pol η crystals at pH 7.0 and then soaked the crystals in reaction buffer containing Mg^2+^ or Mn^2+^ (Supplementary Fig. 1a). After soaking the Pol η crystals in 1 mM Mg^2+^ reaction buffer for 40 s, K^1+^ dissociated from the Me^2+^_A_ site, and Ca^2+^ started to dissociate from the Me^2+^_B_ site, leaving 60% and 50% of Mg^2+^ in the Me^2+^_A_ and Me^2+^_B_ sites, respectively (Fig. 2a). Binding of the Mg^2+^_A_ induced partial primer 3′-OH alignment. Around 35% of the primer end resided in the aligned down conformation, with the 3′-OH existing 2.5 Å from the Mg^2+^_A_, 0.5 Å longer than the perfect coordination distance for Mg^2+^^50^. The 3′-OH was ~175° in-line and 3.6 Å away from the α-phosphate, which may be too far for initiating a nucleophilic attack. In contrast, for the Pol η crystals with the correct substrate that were soaked in the same condition for the same reaction time, all of the primer end was found in the down conformation, existing 2.2 Å away from the Mg^2+^_A_ as well as 3.2 Å away and ~174° in-line from the α-phosphate.

We next tracked the electron density of the product phosphate and the Me^2+^_C_ during catalysis. After soaking for 80 s in 1 mM Mg^2+^, the primer’s position moved 0.2 Å closer to the α-phosphate (Fig. 2b). Surprisingly, the Mg^2+^_C_ was observed while no density was detected for the converted product. The Mg^2+^_C_ at this timepoint was 2.5 Å and 2.6 Å away from the pro-Sp phosphate oxygen and α-β-phosphate bridging oxygen, respectively, with a bond-angle of 70° (Fig. 2e). In addition, four water molecules completed the octahedral coordination for the Mg^2+^_C._ We assigned the Mg^2+^_C_ with an occupancy of 0.15, at which the B-factor of the surrounding atoms was similar to that of the Mg^2+^_C_. After soaking for 140 s, compared to the 80 s structure, the primer moved 0.2 Å closer to the substrate α-phosphate (3.2 Å away) (Fig. 2c) and the Mg^2+^_C_ moved 0.5 Å closer to the product oxygen (2.2 Å and 2.3 Å away from the product oxygen and pyrophosphate (PP_i_) oxygen respectively) (Fig. 2f and Supplementary Fig. 5a). In conjunction with the shift of the Mg^2+^_C_, density for the product phosphate appeared. With a longer incubation time of 250 s, densities corresponding to both the new bond and the Mg^2+^_C_ increased, and the Mg^2+^_C_ remained well coordinated by the product oxygen and PP_i_ oxygen (Fig. 2d). Just like in correct nucleotide incorporation, the Mg^2+^_C_ density correlated well with the density of the product phosphate (*R*^2^ = 0.98) (Fig. 2g). It is important to note that the first detected density for the Mg^2+^_C_ did not cross the origin in the Mg^2+^_C_-bond formation correlation plot, as it appeared before any product was detected.

Previous studies with Pol η^4,48^, Pol β^5,6^, *Bacillus*^51^, and *Geobacillus*^35^ DNA Pol I have revealed that the incorporated nucleotide moves to the primer position after nucleotidyl transfer and PP_i_ dissociation. For three crystals soaked in 1 mM Mg^2+^ reaction buffer for 300 s, moderate density was detected in the spacious region of Pol η’s active site away from the catalytic residues. The product state translocated almost 20 Å and flipped out of the active site to reside in close proximity to an alanine residue in the neighboring unit cell (Fig. 2h). In place of the product state, water molecules were detected within the active site.

### In crystallo observation of misincorporation by Pol η in the presence of Mn^2+^

**Fig. 3:**
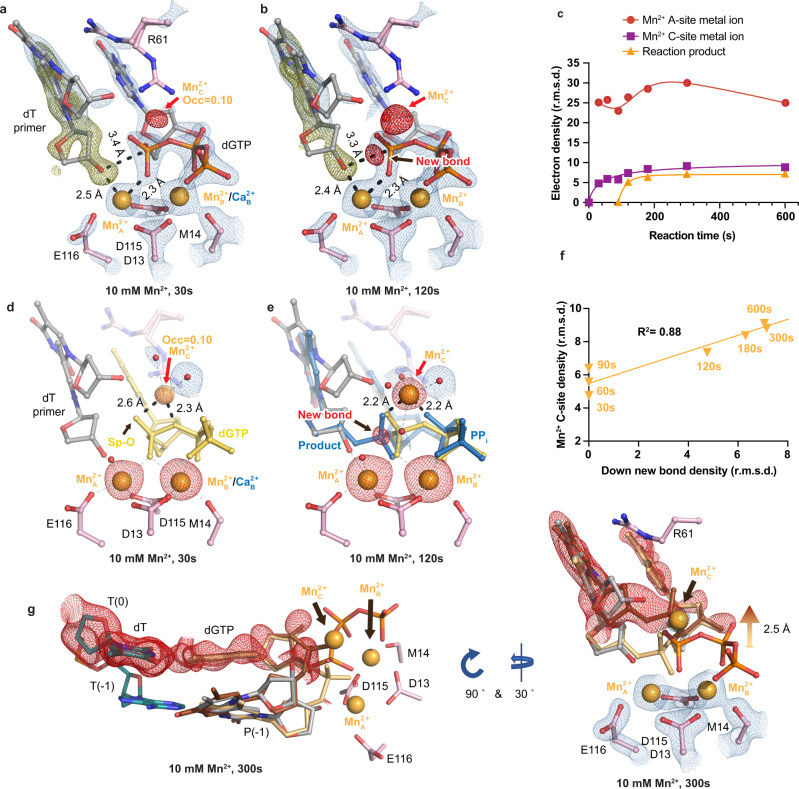
In crystallo visualization of Pol η misincorporation with Mn^2+^. **a**, **b** Structures of Pol η during in crystallo catalysis after 10 mM Mn^2+^ soaking for 30 s (**a**) and 120 s (**b**). The 2F_o_-F_c_ map for the primer up conformation (blue) was contoured at 1.2 σ. The 2F_o_-F_c_ map for the Me^2+^_A_, Me^2+^_B_, dGTP, and catalytic residues (blue) were contoured at 2.5 σ in (**a**–**c**). The F_o_-F_c_ omit map for the down conformation of the primer (green) was contoured at 3.3 σ in (**a**–**c)**. The F_o_-F_c_ omit map for all the Mn^2+^_C_, and newly formed bond (red) was contoured at 4 σ. **c**Timescale of Mn^2+^_A_, Mn^2+^_C_ binding and reaction product in crystallo with Mn^2+^. **d**, **e** Observation of the Mn^2+^_C_ after 10 mM Mn^2+^ soaking for 30 s (**d**) and 120 s (**e**). **f** Correlation (*R*^2^) between the newly formed bond and the Mn^2+^_C_ binding. In **d**, **e**, the 2F_o_-F_c_ map for the water molecules that were coordinated by the Mn^2+^_C_ was contoured at 0.7 σ. The F_o_-F_c_ omit map for the Mn^2+^_C_, new bond, and Mn^2+^_A_ and Mn^2+^_B_ was contoured at 4 σ. **c**, **f** The data points represent singular measurements for r.m.s. density values of electron density. **g** Alternative product state of Pol η in crystallo with 10 mM Mn^2+^ at 300 s. The 2F_o_-F_c_ map for the primer up conformation, primer down conformation, Me^2+^_A_, Me^2+^_B_, Me^2+^_C_, and dGTP (blue) was contoured at 2 σ. The F_o_-F_c_ omit map for the up conformation of the product state (red) was contoured at 3 σ.

We also investigated the misincorporation process supported by error-prone Mn^2+^. After soaking in 10 mM Mn^2+^ for 30 s, Mn^2+^ occupied 65% and 80% at the Me^2+^_A_ and Me^2+^_B_ sites, respectively. At this point, 40% of the primer end existed in the down conformation, with the 3′-OH 2.5 Å away from the Mn^2+^_A_ and 3.4 Å away to the α-phosphate (Fig. 3a). The distances between the Me^2+^_A_ and the 3′-OH were greater than the ideal coordination distances with both Mg^2+^ and Mn^2+^^50^, indicating that 3′-OH alignment during misincorporation was unfavorable.

Similar to the Mg^2+^ time-resolved results, after 30 s of soaking in 10 mM Mn^2+^, the Mn^2+^_C_ was observed (Fig. 3a, d). The Mn^2+^_C_ was coordinated by four water molecules and the incoming dGTP, but the Mn^2+^_C_ remained too far from the phosphate oxygen and bridging oxygen (2.6 Å and 2.3 Å away). We assigned the Mn^2+^_C_ with an occupancy of 0.10, and the B-factor of the surrounding atoms after refinement was similar to that of the Mn^2+^_C_. Furthermore, longer soaking times of 60 s and 90 s in Mn^2+^ resulted in larger densities for the Mn^2+^_C_, of which were assigned with 0.13 and 0.15 occupancies, respectively, but the product density was still not observed. After 120 s of Mn^2+^ soaking, small amount of product was observed, and the Mn^2+^_C_ was found to be 0.5 Å closer towards the phosphate oxygen, compared to the 90 s structure (Fig. 3b, e and Supplementary Fig. 5b, c). After 180 s, both Mn^2+^_C_ and new bond densities grew larger, indicating more Mn^2+^_C_ binding and product formation (Fig. 3c). Just like for the reaction in Mg^2+^, the Mn^2+^_C_ density correlated well with the density of the product phosphate (*R*^2^ = 0.88) (Fig. 3f) and did not cross the origin in the Mn^2+^_C_-bond formation correlation plot. Contrasting with the flipped-out product state with Mg^2+^, a fraction of the product shifted 2.5 Å away from the Mn^2+^_A_ and Mn^2+^_B_ sites (Fig. 3g) and remained in a wobble base-pair with the template dT after 300 s soaking.

### The Me^2+^_C_ is essential for misincorporation

**Fig. 4:**
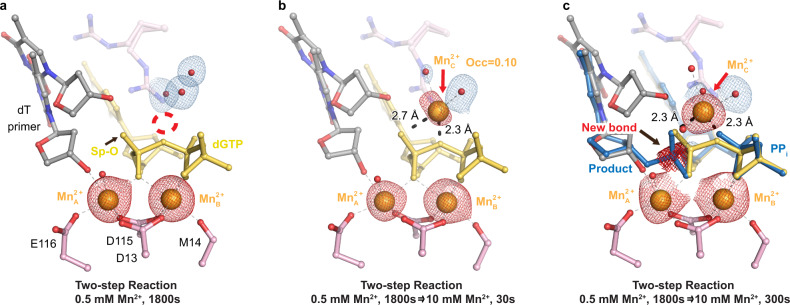
The Me^2+^_C_ is essential for misincorporation. Structures of Pol η in crystallo after 0.5 mM Mn^2+^ soaking (**a**) and subsequent 10 mM Mn^2+^ soaking for 30 s (**b**) and 300 s (**c**). The 2F_o_-F_c_ map for all the water molecules coordinating the Me^2+^_C_ was contoured at 0.7 σ. The F_o_-F_c_ omit map for all Mn^2+^ ions and new bond density (red) was contoured at 3 σ.

The Me^2+^_C_ had been shown to be essential for correct nucleotide incorporation in previous time-resolved reports^34^. Because our time-resolved results illustrated that the Me^2+^_C_ appeared in the reactant states for both Mg^2+^ and Mn^2+^, we wanted to confirm if the Me^2+^_C_ was essential for misincorporation. In previous experiments, the Me^2+^_A_ and Me^2+^_B_ binding could be separated from Me^2+^_C_ binding and catalysis by soaking the Pol η crystals in low concentrations of Mn^2+^^34^. Soaking Pol η crystals in 0.5 mM Mn^2+^ buffer for 30 min allowed Mn^2+^ to saturate the Me^2+^_A_ and Me^2+^_B_ sites to ~80% occupancy (Fig. 4a). At this stage, the majority (50%) of the primer was aligned with the substrate. The primer 3′-OH was 3.1 Å away from the α-phosphate and the 3′-OH was 2.3 Å away from the Mn^2+^_A_. Yet, neither the Mn^2+^_C_ nor product densities were observed. The absence of product formation implied that something essential for catalysis was missing. To test if the Mn^2+^_C_ was the missing component, a two-step reaction was performed by first soaking the crystal in 0.5 mM Mn^2+^ for 30 min and then in reaction buffer that contained 10 mM Mn^2+^ (Supplementary Fig. 2b). After 30 s of soaking in 10 mM Mn^2+^, the Mn^2+^_C_ density was detected before product formation and assigned with an occupancy of 0.10 (Fig. 4b); however, the Mn^2+^_C_ was not in an ideal octahedrally coordinated complex, existing 2.7 Å and 2.3 Å away from the substrate pro-Sp oxygen and α-β-phosphate bridging oxygen, respectively. After 300 s of soaking, both the product and the Mn^2+^_C_ were clearly present. The Mn^2+^_C_ density was 2.3 Å away from both the phosphate oxygen and PP_i_ oxygen, forming an octahedrally coordinated complex (Fig. 4c and Supplementary Fig. 5d).

### Correlation of Me^2+^_A_ binding with primer 3′-OH-substrate alignment

**Fig. 5:**
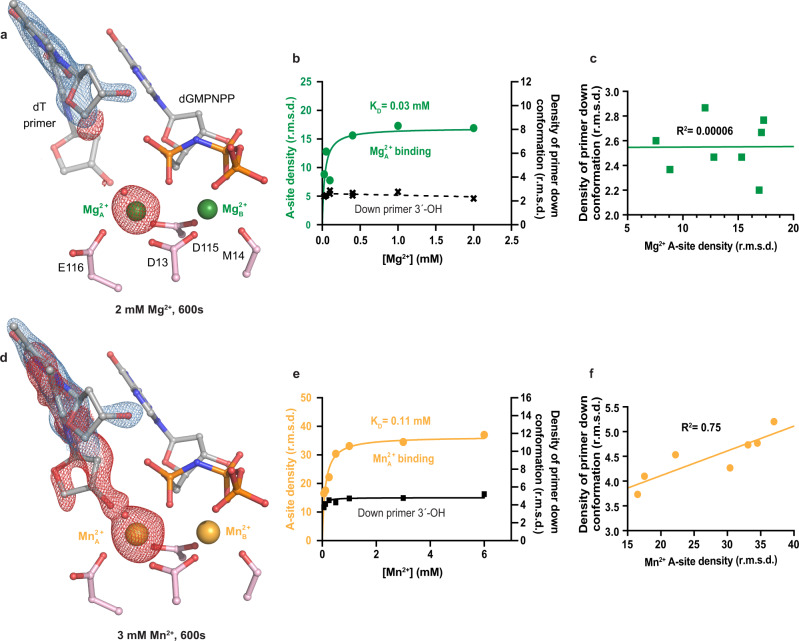
Titration of the Me^2+^_A_ on primer 3′-OH alignment. **a** Structure of Pol η:dGMPNPP with 2.0 mM Mg^2+^. **b** Plot showing Mg^2+^_A_ density and omit map electron density for the down primer with Mg^2+^ concentration. **c** Correlation (*R*^2^) between the Mg^2+^_A_ density and omit map electron density for the down primer. **d** Structure of Pol η:dGMPNPP with 3.0 mM Mn^2+^. **e** Plot showing Mn^2+^_A_ density and omit map electron density for the down primer with Mn^2+^ concentration. **f** Correlation (*R*^2^) between the Mn^2+^_A_ density and omit map electron density for the down primer conformation. The 2F_o_-F_c_ map for the primer up conformation (blue) was contoured at 2 σ in (**a**) and 1.5 σ in (**d**). The F_o_-F_c_ omit map for both the Me^2+^_A_ and primer down conformation (red) were contoured at 3 σ. All data points shown in the plot were collected at *t*  =  600 s and represent singular measurements.

Our time-resolved results suggested that primer 3′-OH alignment and Me^2+^_A_ metal binding were key steps in misincorporation. However, due to conformational changes associated with product formation, it was difficult to confidently correlate primer 3′-OH alignment and Me^2+^_A_ binding. To further investigate the role of the Me^2+^_A_, we used a nonhydrolyzable analog of the incorrect substrate dGMPNPP and titrated the Me^2+^_A_ in crystallo with different concentrations of Mg^2+^ or Mn^2+^ (Supplementary Fig. 2c). Pol η crystals complexed with dGMPNPP were soaked in Mg^2+^ reaction buffer ranging from 0.025 to 2.0 mM Mg^2+^ for 10 min. We monitored the density of the Me^2+^_A_ and the primer in the aligned conformation. Although the Mg^2+^_A_ bound with a K_D_ of 0.03 mM, rendering an omit F_o_-F_c_ map failed to produce sufficient densities that could be confidently assigned with the down conformation of the primer sugar moiety for a Pol η crystal that was soaked in 2 mM Mg^2+^ for 600 s (Fig. 5a). Near the primer termini, only small spherical densities possibly corresponding to water molecules appeared in the void. We suspected that the occupancy of the down conformation of the primer was too low to be assigned (Fig. 5b, c). We conducted the similar protocol with Mn^2+^ reaction buffer ranging from 0.06 mM to 6.0 mM Mn^2+^ for 10 min, and observed that the Me^2+^_A_ site could be fully saturated with a K_D_ of 0.11 mM. In contrast with the in crystallo Mg^2+^_A_ titration results, 30–40% of the primer 3′-OH was aligned for a Pol η crystal that was soaked in 3 mM Mn^2+^ for 600 s (Fig. 5d). A moderate correlation (*R*^2^ = 0.75) for the Me^2+^_A_ and primer alignment was detected (Fig. 5e–f).

## Discussion

### The dynamic reaction process of misincorporation

**Fig. 6:**
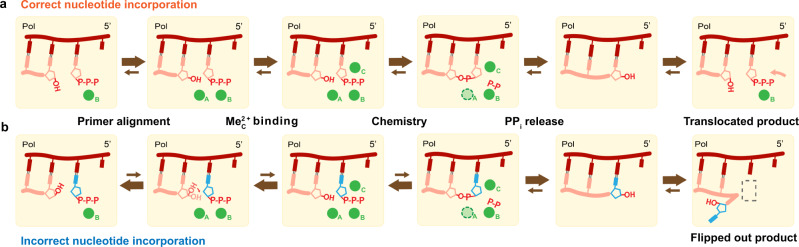
Kinetic model of DNA polymerase catalysis. Proposed kinetic model during DNA polymerase correct nucleotide incorporation (**a**) and misincorporation (**b**). During correct nucleotide incorporation, the primer 3′-OH is well-aligned by the Me^2+^_A_, and the reaction proceeds right after Me^2+^_C_ binding. In contrast, during incorrect nucleotide incorporation, due to reduced base-stacking, primer 3′-OH alignment is unfavored, which would lead to incorrect dNTP dissociation. Error-prone Mn^2+^_A_ can drive 3′-OH alignment and once the Me^2+^_C_ binds, catalysis ensues.

In this study, we captured intermediate states pre-, intra-, and post-reaction at atomic resolution, elucidating the reaction process of the misincorporation of dGTP across dT by Pol η (Supplementary Movie 1). The process of misincorporation after DNA binding begins with a bound Me^2+^_B_-complexed dGTP inside the polymerase active site. At this point, the majority of the primer 3′-OH exists in the up conformation not aligned with the α-phosphate of dGTP, and most of the R61 sidechain is interacting with the wobble base-pair (Fig. 1d, e). Upon Me^2+^_A_ binding, part of the primer 3′-OH shifts downwards and approaches the substrate α-phosphate (Figs. 2a and 3a). Meanwhile, the Me^2+^_C_ binds but remains far from the substrate pro-Sp oxygen (Figs. 2e and 3d). Downward movement of the Me^2+^_C_ and nucleophilic attack then ensue with new-bond formation (Fig. 3b, e). In the presence of Mg^2+^, the product base flips out of the active site (Fig. 2h). This flip-out product may then inhibit primer extension after misincorporation. While in the presence of Mn^2+^, the product is stabilized within the active site (Fig. 3g). In similarity to correct nucleotide incorporation and other time-resolved studies, large movements in the substrate nucleotide were not observed^4,5,34^. Instead, intermediate snapshots revealed that primer 3′-OH alignment was greatly affected between correct and incorrect nucleotide incorporation. This could be attributed to unfavorable base stacking, stemming from the geometry of the dGTP:dT wobble base-pair^48^. Despite this, primer alignment mediated by Me^2+^_A_ binding was shown not to be rate-limiting as revealed by the two-step reaction, in which primer alignment occurred before the second Mn^2+^ soaking step. Perturbation in primer alignment due to buckled base-pairing has also been captured in other polymerases such as the X-family Pol β, suggesting that 3′-OH misalignment is a common mechanism in preventing polymerase misincorporation^52^. Misalignment of the 3′-OH to the Me^2+^_A_ and substrate might favor incorrect nucleotide dissociation versus nucleotidyl transfer and contribute to substrate discrimination, as suggested by Tsai, Johnson, Goodman, and co-workers^25,29–31^ (Fig. 6). On the other hand, the substrate was already bound inside Pol η’s active site in our crystal soaking set-up. Consequentially, conformational changes before and during substrate binding and after product release were not captured. Thus, we cannot rule out the possibility of other rate-limiting conformational steps during polymerase catalysis.

### The Me^2+^_C_ appears before product formation

Since the Me^2+^_C_’s first visualization, the role of the Me^2+^_C_ in polymerase catalysis has been extensively debated^3,5,6^. Similar to correct nucleotide incorporation, we observed that Me^2+^_C_ binding was correlated with product formation (Figs. 2g and 3f) and reaction did not occur without Me^2+^_C_ binding (Fig. 4). Without further details, one could interpret that the Me^2+^_C_ only binds to and stabilizes the product state and shifts the equilibrium towards product formation. In addition, the Me^2+^_C_ was only observed after product formation in Pol β and was missing from the mismatch intermediate structures^5^. Thus, it was proposed that the Me^2+^_C_ only stabilized the product state and was not involved in driving the forward reaction. Conflicting results were reported from computational simulations^53–55^. Some have suggested that Me^2+^_C_ binding does not lower the energy barrier required for catalysis but rather aids in pyrophosphate release^53^ while others have suggested that Me^2+^_C_ binding does stabilize the transition state and significantly lowers the barrier^56^. The most important detail in addressing the debate lies in the timing of Me^2+^_C_ binding. To be catalytic, the Me^2+^_C_ must bind before product formation. We here, observed the Me^2+^_C_ in the reactant state before any product formation (Figs. 2b, e, 3a, d, and 4b). This could be because R61 vacated the Me^2+^_C_ binding site to stabilize the wobble base-pair; however previous studies have shown that the timing of Me^2+^_C_ binding was unchanged in the R61A mutant versus WT Pol η^34^. Another reason could be that additional interactions by the primer up conformation helped to trap the Me^2+^_C_; however, the up primer 3′-OH existed ~3.5 Å away from the Me^2+^_C_, which may be too far for direct coordination. During its initial binding, Me^2+^_C_ coordination was not an optimal octahedrally coordinated complex, as it existed 2.6–2.7 Å away from the substrate pro-Sp oxygen and α-β-phosphate bridging oxygen with a bond angle of 70°. Only when the Me^2+^_C_ moved to its ideal coordination, did we see the product density. These results support that the Me^2+^_C_ is catalytic and drives nucleotidyl transfer by stabilizing the transition state. Observation of a transiently bound metal ion within the active sites of metalloenzymes such as the Me^2+^_C_ in Pol η has become more common with the advent of time-resolved crystallography. The increasing pervasiveness of such observations in Pol η^4,34^, Pol β^5,6,57,58^, and most recently, Pol μ^59^, RNaseH^7^, and Endonuclease V nucleases^60^, suggests that catalysis induced by transiently bound metal ions might be more universal than originally thought.

### The Me^2+^_A_ is responsible for primer 3′-OH alignment and primer 3′-OH alignment is key in misincorporation

In this study, we showed that primer alignment was perturbed during misincorporation. This primer movement has largely been overlooked, as early ternary complex structures of DNA polymerases were complexed with dideoxynucleosides at the primer termini in order to inhibit the reaction. Without a functional 3′-OH group, many of these structures failed to capture the Me^2+^_A_ or instead showed drastic changes in its position^15,61^. Nevertheless, the Me^2+^_A_ has been thought to bridge the primer 3′-OH with the substrate α-phosphate into phosphoryl-transfer proximity and decrease the pKa of the primer 3′-OH group. Improper 3′-OH-Me^2+^_A_ alignment was first captured in Pol β^52^ with nonhydrolyzable dNTP analogs, which revealed significant reorganizations in primer alignment. In 2012, the polymerase reaction for correct nucleotide incorporation was observed in crystallo^4^. Significant movement of the primer 3′-OH conformation and sugar pucker together with the binding of a water molecule proceeded nucleotidyl transfer^4,34^. For incorrect nucleotide incorporation, primer misalignment was observed in the time-resolved studies of Pol β, but intermediate structures that led to primer alignment were not captured^5^. Perturbed 3′-OH alignment was also observed in the extension of phenanthriplatin DNA damage as well as bypass of 8,5′-cyclo-2′-deoxyadenosine lesions^62,63^. Here, we captured primer alignment and correlated it with Me^2+^_A_ binding (Fig. 5e, f). In the time-resolved results, the Me^2+^_A_ was not in its ideal octahedral geometry and its bond distance was far, suggesting major barriers in aligning the 3′-OH. Mn^2+^ was shown to be more optimal than Mg^2+^ at aligning the primer, explaining why Mn^2+^ is error-prone in polymerase catalysis. In addition, our results suggested that the nonhydrolyzable analog dGMPNPP, although similar, is not identical to dGTP in promoting 3′-OH alignment. This is possibly due to the different electrostatic nature stemming from the nitrogen atom. Our studies again highlighted the power of observing the catalytic process with native substrates through time-resolved crystallography.

## Methods

### Protein expression and purification

Wild-type human polymerase η (Pol η) (residues 1–432) was cloned into a modified pET28p vector with a N-terminal 6-histidine tag and a PreScission Protease cleavage site as described^47^. For protein expression, this Pol η plasmid was transformed into BL21 DE3 *E. coli* cells. When the optical density of the *E. coli* cells reached 0.8, isopropyl ß-D-1-thiogalactopyranoside (IPTG) was added to a final concentration of 1 µM IPTG. After 20 h. (16 °C) of induction, the cell paste was collected via centrifugation and re-suspended in a buffer that contained 20 mM Tris (pH 7.5), 1 M NaCl, 20 mM imidazole, and 5 mM ß-mercaptoethanol (BME). After sonification, Pol η was loaded onto a HisTrap HP column (GE Healthcare), which was pre-equilibrated with a buffer that contained 20 mM Tris (pH 7.5), 1 M NaCl, 20 mM imidazole, and 5 mM BME. The column was washed with 300 mL of buffer to remove non-specific bound proteins and was eluted with buffer that contained 20 mM Tris (pH 7.5), 1 M NaCl, 300 mM imidazole, and 3 mM dithiothreitol (DTT). The eluted Pol η was incubated with PreScission Protease to cleave the N-terminal 6-histidine-tag. Afterwards, Pol η was buffer-exchanged and desalted to 20 mM 2-(N-morpholino)ethanesulfonic acid (MES) (pH 6.0), 250 mM KCl, 10% glycerol, 0.1 mM ethylenediaminetetraacetic acid (EDTA), and 3 mM DTT and was loaded onto a MonoS 10/100 column (GE Healthcare). The protein was eluted with an increasing salt (KCl) gradient. Finally, Pol η was cleaned with a Superdex 200 10/300 GL column (GE Healthcare) with a buffer that contained 20 mM Tris (pH 7.5), 450 mM KCl, and 3 mM DTT.

### DNA synthesis assay

The nucleotide incorporation activity was assayed by the following: The reaction mixture contained 2.5-8 nM Pol η, 5 µM DNA, 0–400 µM dNTP (either dATP or dGTP), 100 mM KCl, 50 mM Tris (pH 7.5), 5 mM MgCl_2,_ 3 mM DTT, 0.1 mg/mL bovine serum albumin, and 4% glycerol. The incorporation assays were executed using DNA template (5′-GAG TCA TGT TTA CGC TAG GCA C-3′) and 5′-fluorescein- labeled primer (5′-GTGCCTAGCGTAA-3′). Reactions were conducted at 37 °C for 5 min and were stopped by adding formamide quench buffer to the final concentrations of 40% formamide, 50 mM EDTA (pH 8.0), 0.1 mg/ml xylene cyanol, and 0.1 mg/ml bromophenol. After heating to 97 °C for 5 min and immediately placing on ice, reaction products were resolved on 22.5% polyacrylamide urea gels. The gels were visualized by a Sapphire Biomolecular Imager and quantified using the built-in software. Quantification of K_cat_, K_M_, V_Max_ and fitting and graphic representation were executed by Graph Prism. Source data of urea gels are provided as a Source Data file.

### Crystallization

Pol η was concentrated to 300 µM in buffer that contained 20 mM Tris (pH 7.5), 0.45 M KCl, and 3 mM DTT. Then DNA, dGTP or dGMPNPP, and Ca^2+^ and low salt buffer (20 mM Tris (pH 7.5), and 3 mM DTT) were added to this polymerase solution at the molar ratio of 1:1.2:1:1 for Pol η, DNA, dGTP or dGMPNPP, and Ca^2+^, bringing Pol η’s concentration to 100 µM. Then after this solution was kept on ice for 10 min, more dGTP or dGMPNPP was added to a final concentration of 0.5 mM. DNA template and primer used for crystallization were 5′-CAT GAT GAC GCT-3′ and 5′-AGC GTC AT-3, respectively. All crystals were obtained using the hanging-drop vapor-diffusion method against a reservoir solution containing 0.1 M MES (pH 6.0) and 9–15% (w/v) PEG2K-MME at room temperature within 4 days.

### Chemical reaction in crystallo

The crystals were first transferred and incubated in a pre-reaction buffer containing 0.1 M MES (pH 7.0, titrated by KOH), 100 µM dGTP, and 20% (w/v) PEG2K-MME for 30 min. The chemical reaction was initiated by transferring the crystals into a reaction buffer containing 0.1 M MES (pH 7.0), 20% (w/v) PEG2K-MME, and 0.5-20 mM MgCl_2_ or MnCl_2_. After incubation for a desired time period, the crystals were quickly dipped in a cryo-solution supplemented with 20% (w/v) glycerol and flash- cooled in liquid nitrogen. For the two-step in crystallo reaction, a 30 min incubation step in buffer containing 0.1 M MES (pH 7.0), 0.5 mM MnCl_2_, and 20% (w/v) PEG2K-MME was added before initiation of the reaction. For the A-site in crystallo titration experiment, the crystals were incubated in 0.025–6.0 mM MgCl_2_ or MnCl_2_ for 10 min.

### Data collection and refinement

Diffraction data were collected at 100 K on LS-CAT beam lines 21-D-D, 21-ID-F, and 21-ID-G at the Advanced Photon Source (Argonne National Laboratory). Data were indexed in space group P6_1_, scaled and reduced using XDS^64^. Isomorphous Pol η structures with Mg^2+^ (PDB: 4ECQ, 4ECR or 4ECV)^4^ and Mn^2+^ (PDB: 5K5G and 5L9X) were used as initial models for refinement using PHENIX^65^ and COOT^66^. Initial occupancies were assigned for the substrate, reaction product, PP_i_, Me^2+^_A_, Me^2+^_B_, and Me^2+^_C_, for the ternary ground state, 1 mM Mg^2+^ 300 s, and 10 mM Mn^2+^ 600 s structure following the previous protocol^34^. After there were no significant F_o_-F_c_ peaks and each atom’s B value had roughly similar values to its ligand, we assigned occupancies for the same regions for the timepoints in between. Source data of the electron densities in r.m.s. density are provided as a Source Data file. Each structure was refined to the highest resolution data collected, which ranged between 1.47 and 1.83 Å. Software applications used in this project were compiled and configured by SBGrid^67^. Source data of data collection and refinement statistics are summarized in Supplementary Table 1a–f in the Source data file. All structural figures were drawn using PyMOL (http://www.pymol.org).

### Reporting summary

Further information on research design is available in the Nature Research Reporting Summary linked to this article.

## Supplementary information

Supplementary Information

Description of Additional Supplementary Files

Supplementary Movie 1

Reporting Summary

## Data Availability

The data that support this study are available from the corresponding author upon reasonable request. The coordinates, density maps, and structure factors for all the structures have been deposited in Protein Data Bank (PDB) under accession codes 7U72, 7U73, 7U74, 7U75, 7U76, 7U77, 7U78, 7U79, 7U7A, 7U7B, 7U7C, 7U7D, 7U7E, 7U7F, 7U7G, 7U7I, 7U7J, 7U7K, 7U7L, 7U7R, 7U7S, 7U7T, 7U7U, 7U7V, 7U7W, 7U7X, 7U7Y, 7U7Z, 7U80, 7U81, 7U82, 7U83, and 7U84. Source data are provided with this paper.

## Source data

Source Data
