# Supplementary material for: In crystallo observation of three metal ion promoted DNA polymerase misincorporation
Source: Nat Commun. 2022 Apr 29;13:2346. doi: 10.1038/s41467-022-30005-3 (PMC9054841; doi:10.1038/s41467-022-30005-3)
Supplement: Supplementary file 1 — Supplementary Information [file 41467_2022_30005_MOESM1_ESM.pdf]

Supplementary Information

In Crystallo Observation of Three Metal Ion Promoted DNA Polymerase Misincorporation

Caleb Chang[1], Christie Lee Luo[1], and Yang Gao[1,*]

[1]Department of Biosciences, Rice University, Houston, Texas, 77005, USA

[*]Correspondence: yg60@rice.edu , +1 (713) 348-2619

Supplementary Figures

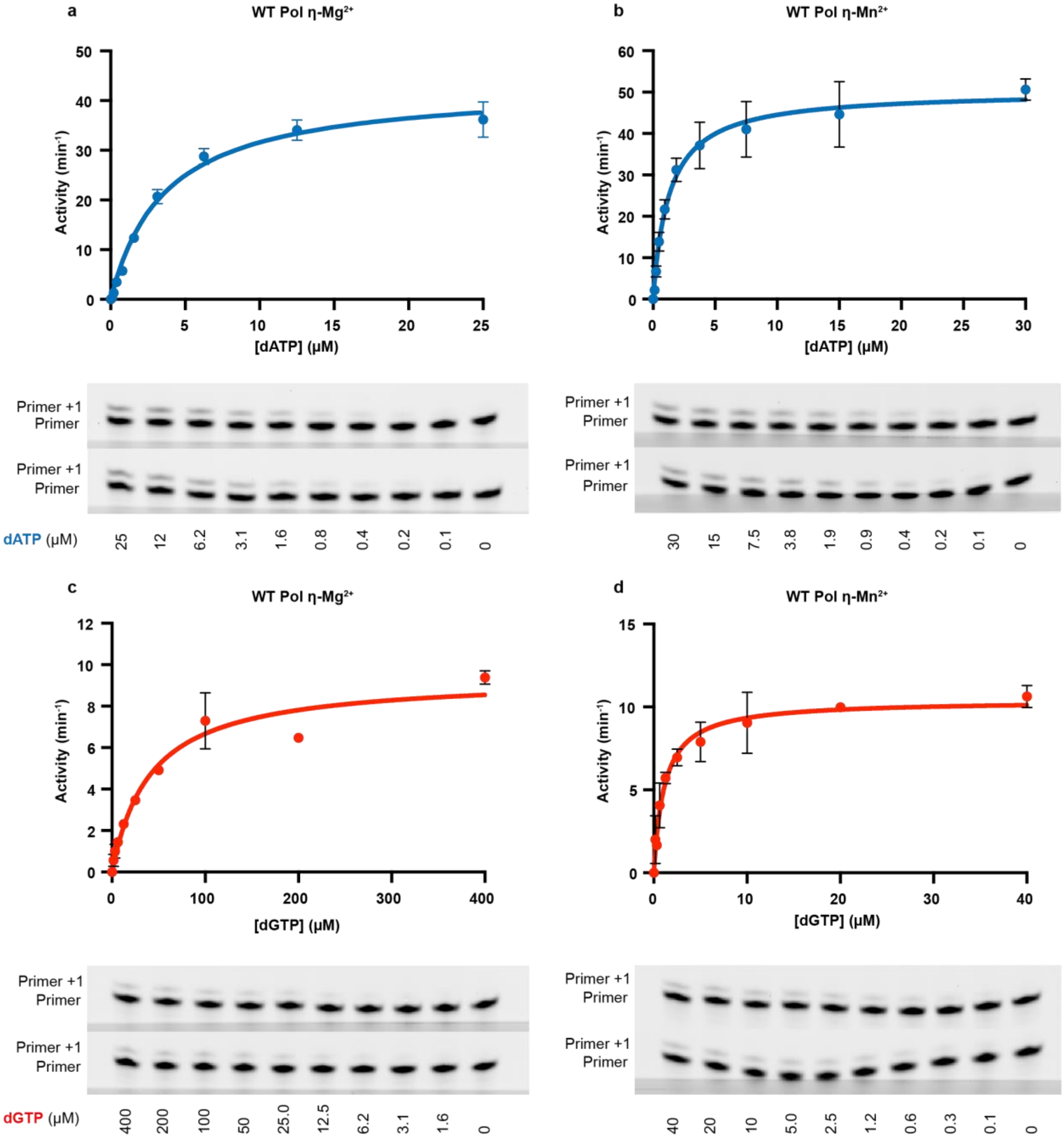

Supplementary Fig. 1: Steady-state single-base nucleotide incorporation assays. **a-b,** Steady-state single-base nucleotide incorporation assays of Pol η in the presence of 5 mM Mg^{2+} for correct incorporation (**a**) and misincorporation (**b**). **c-d,** Steady-state single-base nucleotide incorporation assays of Pol η in the presence of 10 mM Mn^{2+} for correct incorporation (**c**) and misincorporation (**d**). All data points are presented as mean values ± standard deviation of duplicate measurements.

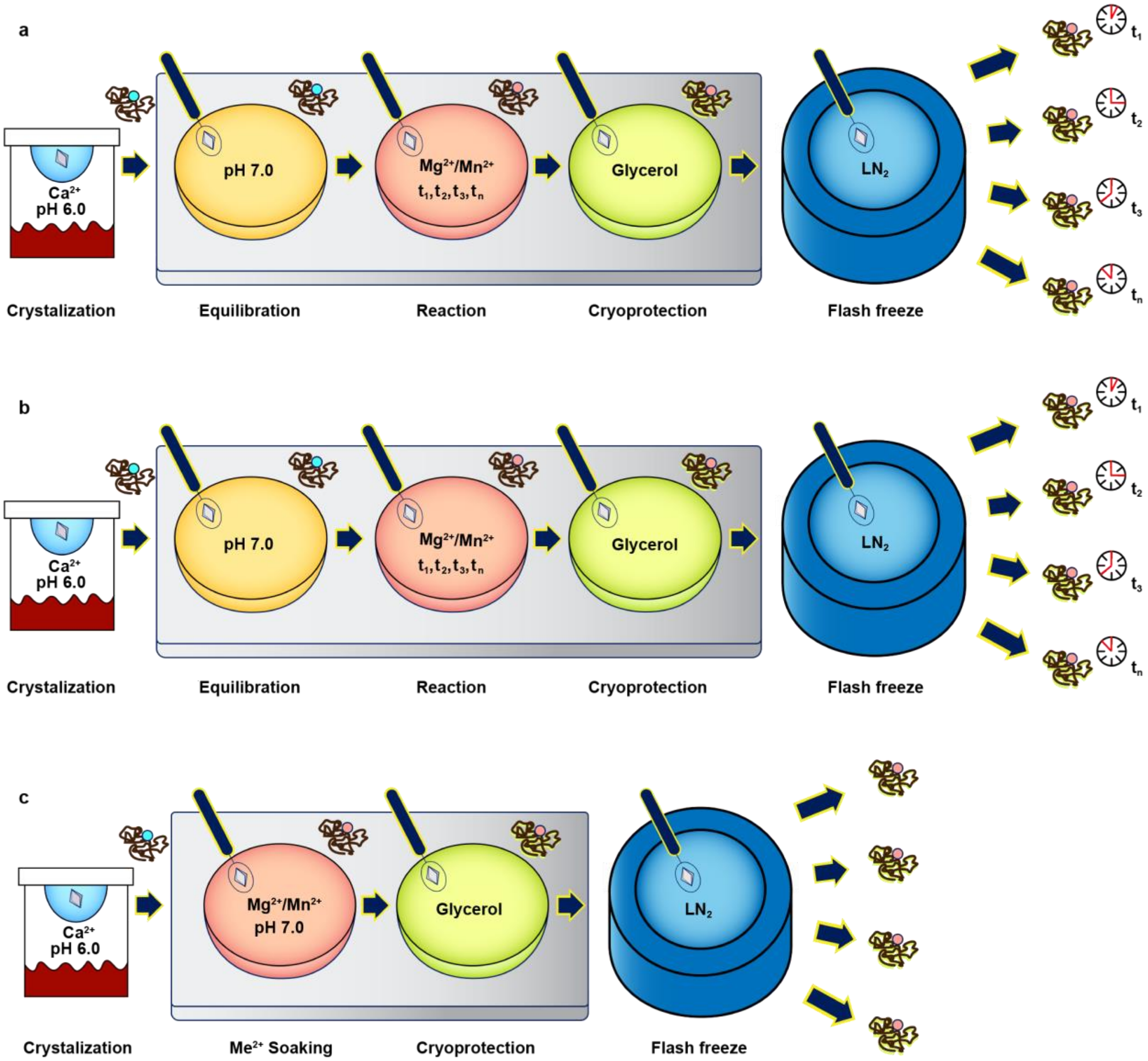

Supplementary Fig. 2: Crystal soaking step-up.
a, Pol η crystal soaking setup for the time-resolved reaction. **b**, Pol η crystal soaking setup for the two-step reaction. **c**, Pol η crystal soaking setup for $Me^{2+}{}_A$ titration.

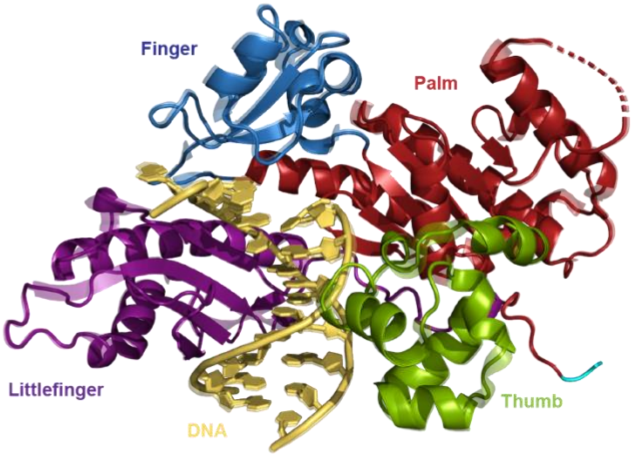

Supplementary Fig. 3: Superposition of correct versus incorrect nucleotide incorporation ternary complexes of Pol η. The DNA is colored in yellow. The palm, finger, thumb, and little finger domains are colored in red, blue, green, and purple, respectively. Faded structures represent the correct incorporation ternary complex.

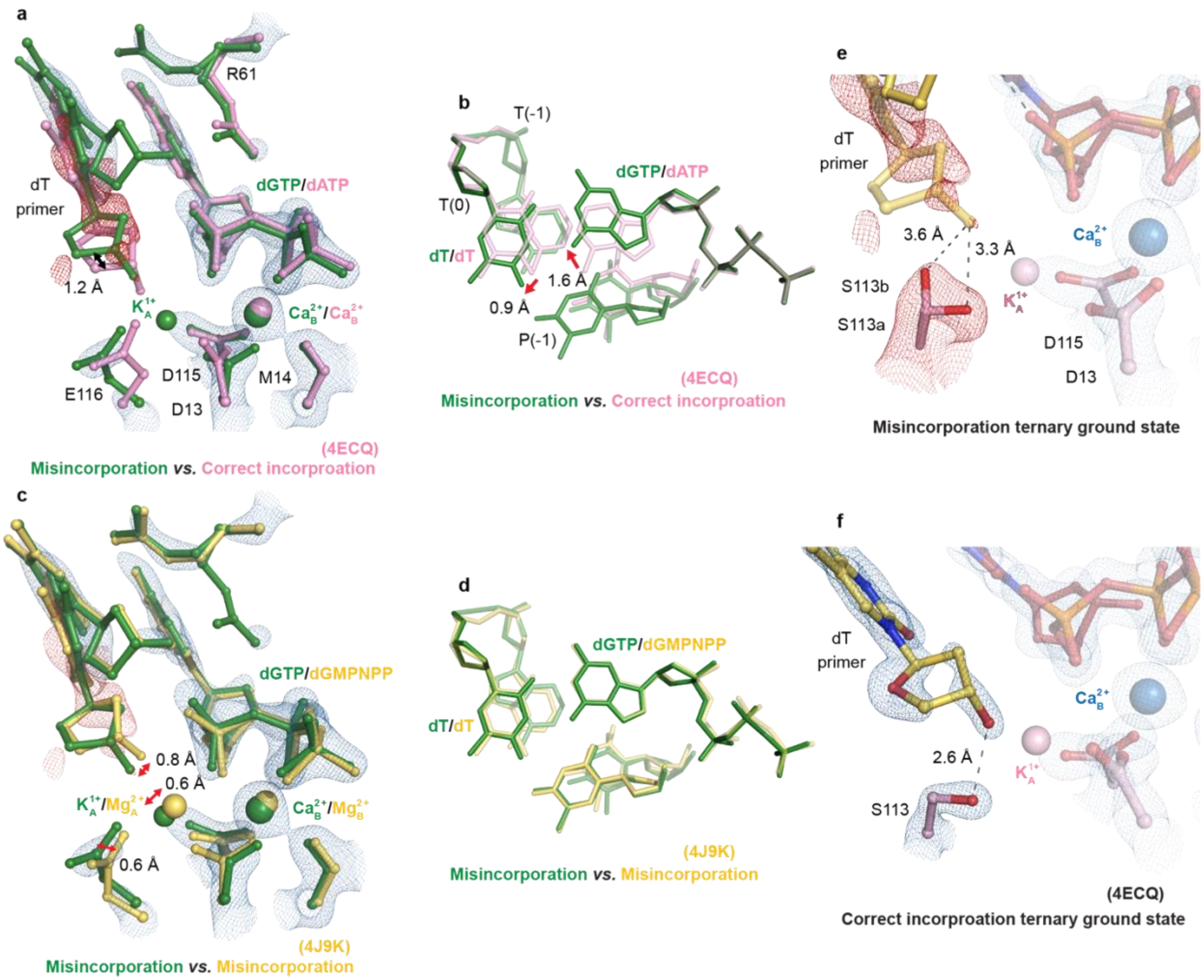

Supplementary Fig. 4: Structural comparison between ground states of correct nucleotide and incorrect nucleotide incorporation.

a, **b,** Structural comparison of incorrect dGTP:dT incorporation (pink) and correct dATP:dT nucleotide incorporation (4ECQ) (green) indicating the differences in 3´-OH alignment (**a**) and base-pairing (**b**). **c**, **d,** Structural overlay of misincorporation structures with dGMPNPP:dT base-pair (4J9K) (yellow) and the ternary ground state with dGTP:dT base-pair dGTP (green) indicating the differences in 3´-OH alignment (**c**) and base-pairing (**d**). **a-d**, All electron density maps apply to the molecule colored in green. **e, f**, Structure of mismatch (**e**) and matched (**f**) Pol η ground state complexed with Ca^{2+}. The $2F_o$-F_c map for everything including primer, $Me^{2+}{}_A$ and $Me^{2+}{}_B$ sites, dGTP, and catalytic residues and S113 in **f** (blue) was contoured at 2 σ. The F_o-F_c omit map for the down conformation of the primer and also S113 in **e** (red) was contoured at 3 σ.

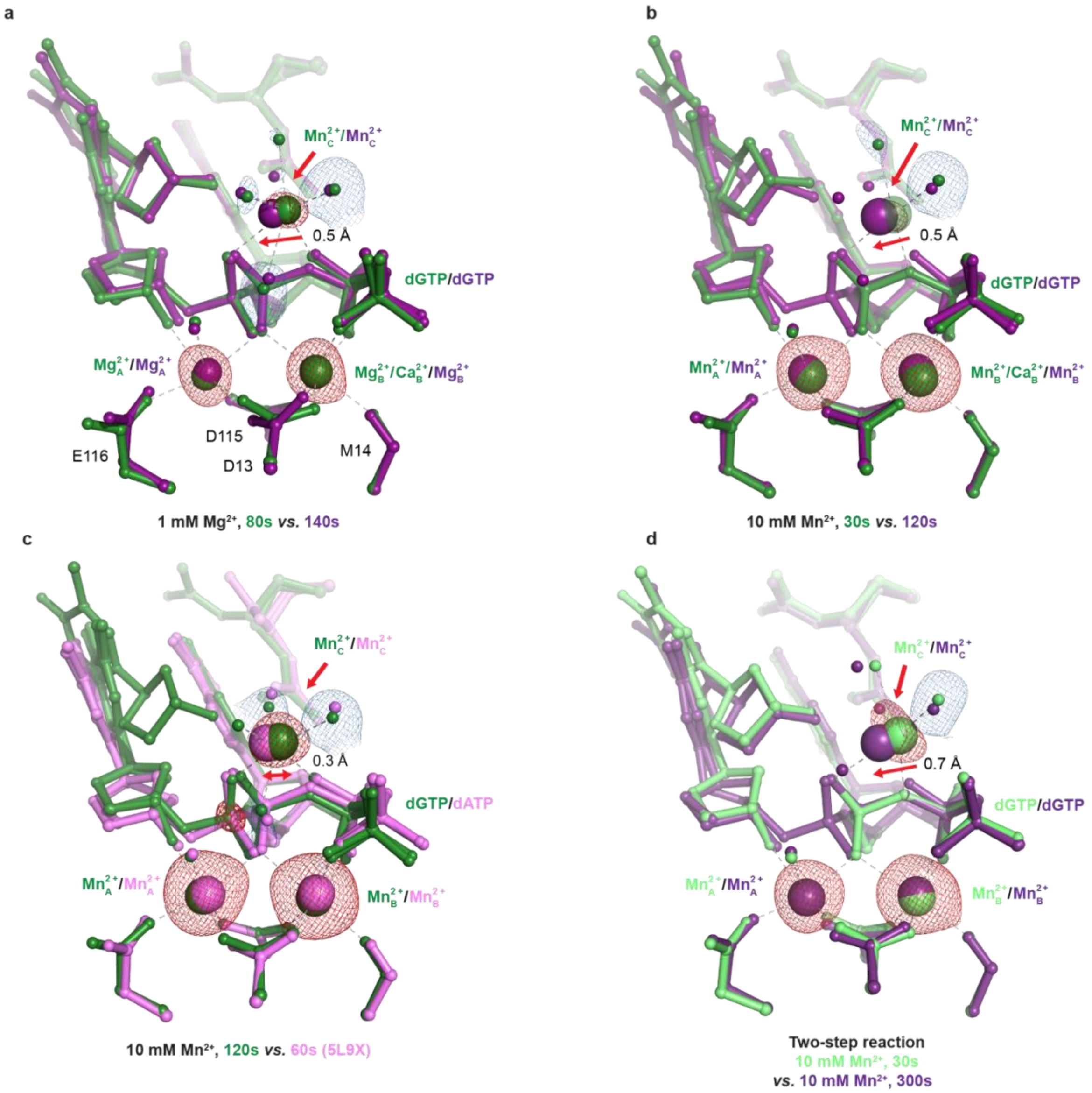

Supplementary Fig. 5: Structural superposition of the Me^{2+}_C.
All electron density maps apply to the molecule colored in green in **a-c** and lime green in **d**. The $2F_o$-F_c maps for all the coordinating water molecules (blue) were contoured at 0.7 σ. The F_o-F_c omit map for the Me^{2+}_A, Me^{2+}_B, Me^{2+}_C and new bond density (red) was contoured at 4 σ in **a, b, c** and 3 σ in **d**.

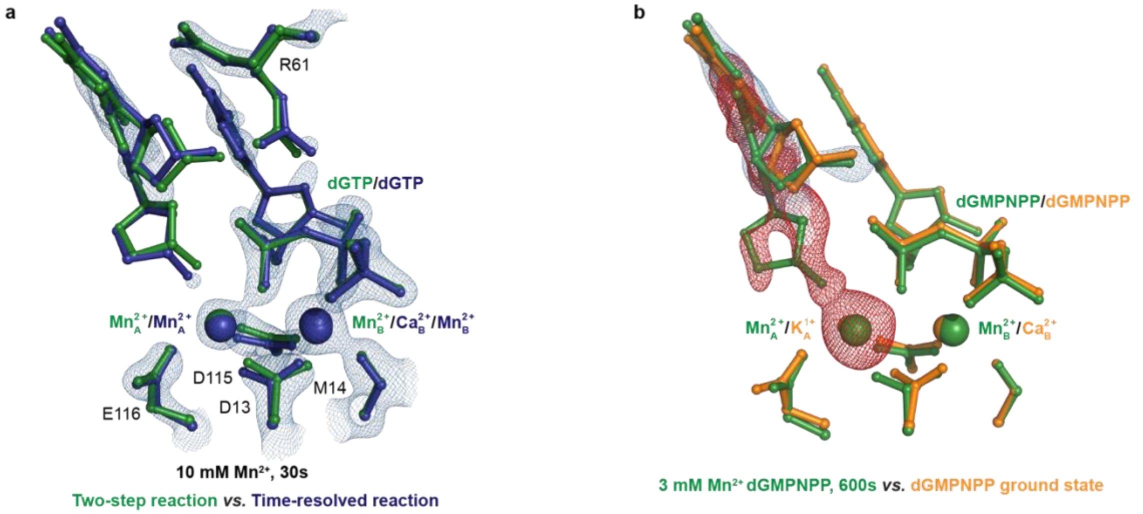

Supplementary Fig. 6: Structural superposition of reaction states and $Me^{2+}{}_A$ *in crystallo* states.

a, Structural comparison between Pol η:dGTP in the two-step reaction (green) and Pol η:dGTP in the time-resolved reaction (navy). The $2F_o$-F_c map for the primer, $Me^{2+}{}_A$, $Me^{2+}{}_B$, catalytic residues, and dGTP (blue) was contoured at 2.0 σ. **b**, Structural comparison between Pol η:dGMPNPP soaked in 3 mM Mn^{2+} for 600s (green) and Pol η:dGMPNPP ground state (orange). The $2F_o$-F_c map for the primer up conformation (blue) was contoured at 1.5 σ. The F_o-F_c omit map for both the $Me^{2+}{}_A$ and primer down conformation (red) was contoured at 3 σ. All electron density maps apply to the molecule colored in green.

Supplementary Tables

Supplementary Table 1: Crystal Diffraction and refinement data.

a Reaction with 1 mM Mg^{2+}

	GS (pH 7.0)	40s	80s	140s
**PDB Code**	**7U72**	**7U77**	**7U78**	**7U79**
**Data collection**				
Wavelength (Å)	0.9787	0.9787	0.9787	0.9787
Space group	$P6_1$	$P6_1$	$P6_1$	$P6_1$
Cell dimensions				
a, b, c (Å)	97.91<br>97.91<br>81.70	98.22<br>98.22<br>82.29	97.74<br>97.74<br>81.76	98.69<br>98.69<br>82.28
α, β, γ (˚)	90, 90, 120	90, 90, 120	90, 90, 120	90, 90, 120
Resolution (Å)[1]	37.6 - 1.53<br>(1.59 - 1.53)	37.8 - 1.58<br>(1.64 - 1.58)	42.0 - 1.6<br>(1.67 - 1.6)	42.3 - 1.69<br>(1.75 - 1.69)
R_{sym} or R_{merge}[1]	0.0478 (1.26)	0.0496 (0.978)	0.0723 (1.79)	0.0811 (2.08)
$I/\sigma I$[1]	23.8 (1.2)	26.4 (1.9)	19.8 (1.2)	20.2 (1.2)
$CC^{1/2}$[1]	1 (0.48)	1 (0.65)	1 (0.41)	1 (0.48)
Completeness (%)	99.9 (99.0)	99.1 (92.7)	99.9 (99.8)	99.8 (98.2)
No. unique reflections[1]	66926<br>(6570)	61140<br>(5732)	57410<br>(5710)	50554<br>(4956)
**Refinement**				
Up primer occ.	0.85	0.70	0.75	0.65
Down primer occ.	0.15	0.30	-	0.10
Up product occ.	-	-	-	-
Down product occ.	-	-	-	0.25
A site occupancy	0.30 K^{1+}	0.60 Mg^{2+}	0.75 Mg^{2+}	0.60 Mg^{2+}
B site occupancy	0.80 Ca^{2+}	0.50 Ca^{2+}<br>0.50 Mg^{2+}	0.30 Ca^{2+}<br>0.70 Mg^{2+}	1.00 Mg^{2+}
C site occupancy	-	-	0.15 Mg^{2+}	0.35 Mg^{2+}
dGTP occupancy	0.80	0.85	0.85	0.60
Product formation	-	-	-	0.25
B-factors				
Me_A/Lig_A [2]	29.1/33.7	25.0/32.4	26.4/33.3	29.4/35.2
Me_B/Lig_B [2]	24.2/26.7	20.7/24.9	21.6/26.8	18.4/27.1
Me_C/Lig_C [2]	-/-	-/-	26.9/30.2	29.4/35.4
Resolution (Å)	1.53	1.58	1.60	1.70
No. reflections	66924 (6570)	61136 (5732)	57405 (5709)	50057 (4997)
R_{work}/R_{free}	0.19/0.22	0.18/0.20	0.19/0.22	0.18/0.22
Wilson B	24.6	24.1	24.5	27.8
Ramachandran				
Favored (%)	97.0	97.9	97.9	97.7
Outlier (%)	0	0	0.47	0.47
R.m.s. deviations				
Bond lengths (Å)	0.009	0.009	0.009	0.01
Bond angles (˚)	1.2	1.2	1.2	1.1

[1]Data in the highest resolution shell is shown in the parenthesis.

[2]B-factor of metal ions and their protein nucleotide ligands.

	200s	250s	300s
**PDB Code**	**7U7A**	**7U7B**	**7U7C**
**Data collection**			
Wavelength (Å)	0.9787	0.9787	0.9787
Space group	*P6₁*	*P6₁*	*P6₁*
Cell dimensions			
a, b, c (Å)	97.92<br>97.92<br>81.67	98.08<br>98.08<br>81.94	98.34<br>98.34<br>82.20
α, β, γ (˚)	90, 90, 120	90, 90, 120	90, 90, 120
Resolution (Å)[1]	37.6 - 1.58<br>(1.64 - 1.58)	37.7 - 1.61<br>(1.67 - 1.61)	42.2 - 1.55<br>(1.61 - 1.55)
R_{sym} or R_{merge}[1]	0.0839 (1.81)	0.0472 (0.529)	0.0453 (0.721)
$I/\sigma I$[1]	20.6 (1.1)	27.2 (3.7)	27.6 (2.4)
$CC^{1/2}$ [1]	1 (0.41)	1 (0.89)	1 (0.79)
Completeness (%)	99.9 (99.8)	99.3 (96.2)	99.0 (90.9)
No. unique reflections[1]	60848<br>(6035)	57668<br>(5559)	64707<br>(5909)
**Refinement**			
Up primer occ.	0.60	0.60	0.50
Down primer occ.	0.10	0.00	0.05
Up product occ.	-	-	-
Down product occ.	0.35	0.40	0.45
A site occupancy	0.60 Mg^{2+}	0.55 Mg^{2+}	0.50 Mg^{2+}
B site occupancy	0.90 Mg^{2+}	1.00 Mg^{2+}	1.00 Mg^{2+}
C site occupancy	0.45 Mg^{2+}	0.55 Mg^{2+}	0.60 Mg^{2+}
dGTP occupancy	0.40	0.35	0.30
Product formation	0.35	0.40	0.45
B-factors			
Me_A/Lig_A [2]	26.8/31.7	26.6/30.9	24.3/29.1
Me_B/Lig_B [2]	17.6/25.0	17.3/25.3	16.5/22.2
Me_C/Lig_C [2]	27.9/30.8	32.2/26.6	32.7/25.9
Resolution (Å)	1.58	1.61	1.55
No. reflections	60843 (6030)	57625 (5559)	64706 (5909)
R_{work}/R_{free}	0.19/0.22	0.18/0.20	0.18/0.21
Wilson B	21.7	21.7	20.8
Ramachandran			
Favored (%)	97.9	97.9	97.9
Outlier (%)	0	0	0
R.m.s. deviations			
Bond lengths (Å)	0.009	0.008	0.008
Bond angles (˚)	1.1	1.1	1.1

[1]Data in the highest resolution shell is shown in the parenthesis.
[2]B-factor of metal ions and their protein nucleotide ligands.

b Reaction with 10 mM Mn^{2+}

	30s	60s	90s	120s
**PDB Code**	**7U7D**	**7U7E**	**7U7F**	**7U7G**
**Data collection**				
Wavelength (Å)	0.9787	0.9787	0.9787	0.9787
Space group	$P6_1$	$P6_1$	$P6_1$	$P6_1$
Cell dimensions				
a, b, c (Å)	98.08<br>98.08<br>82.35	98.00<br>98.00<br>81.94	98.27<br>98.27<br>81.98	97.79<br>97.79<br>82.08
α, β, γ (˚)	90, 90, 120	90, 90, 120	90, 90, 120	90, 90, 120
Resolution (Å)[1]	37.8 - 1.57<br>(1.63 - 1.57)	37.7 - 1.58<br>(1.64 - 1.58)	42.2 - 1.65<br>(1.71 - 1.65)	42.4 - 1.77<br>(1.83 - 1.77)
R_{sym} or R_{merge}[1]	0.0548 (1.07)	0.0789 (1.57)	0.07 (1.52)	0.096 (1.90)
$I/\sigma I$[1]	23.8 (1.6)	19.6 (1.4)	22.3 (1.4)	18.6 (1.3)
$CC^{1/2}$[1]	1 (0.61)	1 (0.49)	1 (0.59)	1 (0.56)
Completeness (%)	99.9 (99.9)	99.9 (99.8)	98.9 (98.8)	99.99 (100)
No. unique reflections[1]	62764<br>(6249)	61140<br>(6061)	53875<br>(5300)	43468<br>(4302)
**Refinement**				
Up primer occ.	0.60	0.60	0.65	0.50
Down primer occ.	0.40	0.40	0.15	0.25
Up product occ.	-	-	0.10	0.10
Down product occ.	-	-	0.10	0.15
A site occupancy	0.65 Mn^{2+}	0.70 Mn^{2+}	0.80 Mn^{2+}	0.80 Mn^{2+}
B site occupancy	0.20 Ca^{2+}<br>0.80 Mn^{2+}	0.90 Mn^{2+}	0.85 Mn^{2+}	0.90 Mn^{2+}
C site occupancy	0.10 Mn^{2+}	0.13 Mn^{2+}	0.15 Mn^{2+}	0.18 Mn^{2+}
dGTP occupancy	0.80	0.80	0.60	0.55
Product formation	-	0.00	0.20	0.25
B-factors				
Me_A/Lig_A [2]	22.7/29.5	23.1/30.2	28.4/30.6	28.6/30.3
Me_B/Lig_B [2]	21.9/24.2	22.4/24.8	23.8/25.7	27.7/27.4
Me_C/Lig_C [2]	25.0/32.7	29.1/28.4	27.6/31.6	29.9/35.2
Resolution (Å)	1.57	1.58	1.65	1.77
No. reflections	62761 (6249)	61136 (6059)	53321 (5300)	43467 (4302)
R_{work}/R_{free}	0.18/0.22	0.18/0.22	0.20/0.25	0.18/0.21
Wilson B	23.3	22.8	26.7	30.0
Ramachandran				
Favored (%)	97.9	97.0	96.5	97.2
Outlier (%)	0.23	0	0.47	0.23
R.m.s. deviations				
Bond lengths (Å)	0.008	0.008	0.009	0.009
Bond angles (˚)	1.1	1.2	1.2	1.2

[1]Data in the highest resolution shell is shown in the parenthesis.
[2]B-factor of metal ions and their protein nucleotide ligands.

	180s	300s	600s
**PDB Code**	**7U7I**	**7U7J**	**7U7K**
**Data collection**			
Wavelength (Å)	0.9787	0.9787	0.9787
Space group	$P6_1$	$P6_1$	$P6_1$
Cell dimensions			
a, b, c (Å)	98.07	98.25	97.78
	98.07	98.25	97.78
	81.81	82.16	81.67
α, β, γ (˚)	90, 90, 120	90, 90, 120	90, 90, 120
Resolution (Å)[1]	37.7 - 1.57	37.8 - 1.58	41.9 - 1.67
	(1.63 - 1.57)	(1.64 - 1.58)	(1.73 - 1.67)
R_{sym} or R_{merge}[1]	0.0546 (1.22)	0.0555 (1.14)	0.0906 (1.83)
I/σ*I*[1]	25.0 (1.4)	23.6 (1.7)	20.9 (1.3)
$CC^{1/2}$ [1]	1 (0.51)	1 (0.64)	1 (0.41)
Completeness (%)	99.9 (98.9)	99.9 (98.8)	99.9 (99.3)
No. unique	62189	61301	51427
reflections[1]	(6110)	(6041)	(5091)
**Refinement**			
Up primer occ.	0.45	0.40	0.35
Down primer occ.	0.20	0.20	0.15
Up product occ.	0.15	0.15	0.20
Down product occ.	0.20	0.25	0.30
A site occupancy	0.70 Mn^{2+}	0.70 Mn^{2+}	0.65 Mn^{2+}
B site occupancy	0.90 Mn^{2+}	0.90 Mn^{2+}	0.90 Mn^{2+}
C site occupancy	0.20 Mn^{2+}	0.25 Mn^{2+}	0.30 Mn^{2+}
dGTP occupancy	0.45	0.40	0.30
Product formation	0.30	0.40	0.45
B-factors			
Me_A/Lig_A [2]	21.3/27.9	19.7/27.2	24.1/28.1
Me_B/Lig_B [2]	20.4/22.5	19.3/21.7	24.7/25.3
Me_C/Lig_C [2]	23.7/31.2	25.4/35.5	32.5/36.1
Resolution (Å)	1.57	1.58	1.67
No. reflections	62183 (6109)	61295 (6041)	51420 (5086)
R_{work}/R_{free}	0.18/0.22	0.18/0.21	0.18/0.22
Wilson B	23.7	23.0	26.8
Ramachandran			
Favored (%)	97.4	97.0	97.2
Outlier (%)	0	0.23	0
R.m.s. deviations			
Bond lengths (Å)	0.009	0.009	0.009
Bond angles (˚)	1.2	1.2	1.2

[1]Data in the highest resolution shell is shown in the parenthesis.
[2]B-factor of metal ions and their protein nucleotide ligands.

c 1 mM Mg^{2+} 300s with alternative product

	1	2	3
**PDB Code**	**7U7L**	-	-
**Data collection**			
Wavelength (Å)	0.9787	0.9787	0.9787
Space group	$P6_1$	$P6_1$	$P6_1$
Cell dimensions			
a, b, c (Å)	98.16<br>98.16<br>81.88	98.44<br>98.44<br>82.13	98.24<br>98.24<br>81.94
α, β, γ (˚)	90, 90, 120	90, 90, 120	90, 90, 120
Resolution (Å)[1]	42.1 - 1.47 (1.52 - 1.47)	42.6 - 1.5 (1.55 - 1.5)	42.5 - 1.7 (1.76 - 1.7)
R_{sym} or R_{merge}[1]	0.053 (0.783)	0.0445 (0.851)	0.0499 (0.547)
I/σ*I*[1]	23.1 (1.5)	25.5 (1.6)	28.8 (2.8)
$CC^{1/2}$ [1]	1 (0.57)	1 (0.48)	1 (0.82)
Completeness (%)	99.9 (98.8)	99.5 (95.6)	99.3 (93.3)
No. unique reflections[1]	75880 (7444)	71910 (6879)	49060 (4559)
**Refinement**			
Product occ.	0.30	0.20	0.20
Alternative product occupancy	0.70	0.80	0.80
Resolution (Å)	1.47	1.50	1.70
No. reflections	75876 (7444)	71899 (6871)	49057 (4559)
R_{work}/R_{free}	0.19/0.22	0.18/0.21	0.18/0.21
Wilson B	19.9	21.0	22.2
Ramachandran			
Favored (%)	97.6	97.4	97.4
Outlier (%)	0	0.24	0.24
R.m.s. deviations			
Bond lengths (Å)	0.008	0.008	0.008
Bond angles (˚)	1.1	1.1	1.1

[1]Data in the highest resolution shell is shown in the parenthesis.
[2]B-factor of metal ions and their protein nucleotide ligands.

d Two-step reaction with 10 mM Mn^{2+}

	0s	30s	90s	300s
**PDB Code**	**7U73**	**7U74**	**7U75**	**7U76**
**Data collection**				
Wavelength (Å)	0.9786	0.9786	0.9786	0.9787
Space group	$P6_1$	$P6_1$	$P6_1$	$P6_1$
Cell dimensions				
a, b, c (Å)	98.15	98.68	98.62	97.66
	98.15	98.68	98.62	97.66
	82.10	82.21	82.06	81.44
α, β, γ (˚)	90, 90, 120	90, 90, 120	90, 90, 120	90, 90, 120
Resolution (Å)[1]	42.1 - 1.56 (1.61 - 1.56)	32.3 - 1.52 (1.57 - 1.52)	37.9 - 1.55 (1.61 - 1.55)	42.3 - 1.69 (1.75 - 1.69)
R_{sym} or R_{merge}[1]	0.0673 (1.67)	0.0406 (0.841)	0.0506 (0.921)	0.0609 (1.72)
$I/\sigma I$[1]	29.4 (1.5)	31.1 (1.9)	23.8 (1.6)	19.7 (1.4)
$CC^{1/2}$ [1]	1 (0.61)	1 (0.78)	1 (0.63)	1 (0.59)
Completeness (%)	100 (100)	99.9 (98.8)	99.6 (96.7)	99.99 (100)
No. unique reflections[1]	63845 (6321)	69770 (6867)	65466 (6351)	49390 (4886)
**Refinement**				
Up primer occ.	0.85	0.60	0.65	0.50
Down primer occ.	0.15	0.40	0.35	0.15
Up product occ.	-	-	-	0.20
Down product occ.	-	-	-	0.15
A site occupancy	0.80 Mn^{2+}	0.50 Mn^{2+}	0.40 Mn^{2+}	0.50 Mn^{2+}
B site occupancy	0.85 Mn^{2+}	0.80 Mn^{2+}	0.75 Mn^{2+}	0.75 Mn^{2+}
C site occupancy	-	0.10 Mn^{2+}	0.20 Mn^{2+}	0.20 Mn^{2+}
dGTP occupancy	0.80	0.70	0.75	0.35
Product formation	-	-	-	0.35
B-factors				
Me_A/Lig_A [2]	21.6/25.0	24.5/33.3	22.6/33.4	28.7/35.5
Me_B/Lig_B [2]	18.2/21.7	22.7/24.9	23.1/27.8	25.7/28.3
Me_C/Lig_C [2]	-	30.28/36.6	36.5/38.8	35.44/40.8
Resolution (Å)	1.56	1.52	1.55	1.69
No. reflections	63842 (6321)	69756 (6860)	65464 (6351)	49388 (4886)
R_{work}/R_{free}	0.18/0.21	0.19/0.22	0.19/0.22	0.18/0.22
Wilson B	23.5	22.9	24.4	29.8
Ramachandran				
Favored (%)	97.9	98.1	97.7	97.2
Outlier (%)	0	0	0.23	0.24
R.m.s. deviations				
Bond lengths (Å)	0.009	0.008	0.009	0.011
Bond angles (˚)	1.2	1.1	1.2	1.3

[1]Data in the highest resolution shell is shown in the parenthesis.
[2]B-factor of metal ions and their protein nucleotide ligands.

e A-site titration with Mg^{2+}

	none	0.025	0.05	0.1
**PDB Code**	**7U7R**	**7U7S**	**7U7T**	**7U7U**
**Data collection**				
Wavelength (Å)	0.9786	0.9786	0.9786	0.9786
Space group	$P6_1$	$P6_1$	$P6_1$	$P6_1$
Cell dimensions				
a, b, c (Å)	97.99<br>97.99<br>81.61	98.45<br>98.45<br>82.20	98.63<br>98.63<br>81.97	98.49<br>98.49<br>82.05
α, β, γ (˚)	90, 90, 120	90, 90, 120	90, 90, 120	90, 90, 120
Resolution (Å)[1]	28.3 - 1.64<br>(1.70 - 1.64)	37.8 - 1.6<br>(1.66 - 1.6)	42.7 - 1.55<br>(1.61 - 1.55)	32.2 - 1.54<br>(1.60 - 1.54)
R_{sym} or R_{merge}[1]	0.0561 (1.57)	0.0565 (1.31)	0.0566 (1.01)	0.0339 (0.984)
I/σ*I*[1]	21.7 (1.3)	24.7 (1.5)	24.3 (1.7)	34.5 (1.7)
$CC^{1/2}$[1]	1 (0.51)	1 (0.59)	1 (0.59)	1 (0.61)
Completeness (%)	99.7 (98.0)	100 (100)	99.0 (91.3)	99.5 (96.4)
No. unique reflections[1]	54007<br>(5291)	59684<br>(5958)	64446<br>(5924)	66491<br>(6416)
**Refinement**				
Up primer occ.	1.00	0.90	0.85	0.80
Down primer occ.	-	0.10	0.15	0.20
A site occupancy	0.20 K^{1+}	0.75 Mg^{2+}	0.75 Mg^{2+}	0.80 Mg^{2+}
B site occupancy	0.80 Ca^{2+}	1.00 Mg^{2+}	1.00 Mg^{2+}	1.00 Mg^{2+}
dGTP occupancy	0.80	0.80	0.80	0.85
B-factors				
Me_A/Lig_A [2]	28.5/33.7	33.0/37.3	24.5/33.4	38.6/35.7
Me_B/Lig_B [2]	22.1/26.1	20.0/27.9	16.5/23.7	18.3/25.3
Resolution (Å)	1.64	1.60	1.55	1.54
No. reflections	54006 (5291)	59681 (5958)	64432 (5919)	66487 (6416)
R_{work}/R_{free}	0.19/0.22	0.19/0.21	0.19/0.20	0.19/0.22
Wilson B	27.7	24.9	21.9	24.3
Ramachandran				
Favored (%)	97.2	97.7	98.1	97.7
Outlier (%)	0.23	0	0	0
R.m.s. deviations				
Bond lengths (Å)	0.008	0.009	0.009	0.009
Bond angles (˚)	1.3	1.2	1.1	1.2

[1]Data in the highest resolution shell is shown in the parenthesis.
[2]B-factor of metal ions and their protein nucleotide ligands.

	0.4	1	2
**PDB Code**	**7U7V**	**7U7W**	**7U7X**
**Data collection**			
Wavelength (Å)	0.9786	0.9786	0.9786
Space group	$P6_1$	$P6_1$	$P6_1$
Cell dimensions			
a, b, c (Å)	98.62	97.97	98.29
	98.62	97.97	98.29
	81.96	81.76	81.95
α, β, γ (˚)	90, 90, 120	90, 90, 120	90, 90, 120
Resolution (Å)[1]	37.9 - 1.65	37.7 - 1.66	28.4 - 1.65
	(1.71 - 1.65)	(1.72 - 1.66)	(1.71 - 1.65)
R_{sym} or R_{merge}[1]	0.0691 (1.74)	0.0581 (1.24)	0.0614 (1.25)
$I/\sigma I$[1]	19.8 (1.4)	26.7 (1.8)	23.2 (1.7)
$CC^{1/2}$[1]	1 (0.55)	1 (0.61)	1 (0.62)
Completeness (%)	100 (99.7)	97.9 (95.0)	99.2 (98.1)
No. unique reflections[1]	54421 (5435)	51398 (4967)	53665 (5308)
**Refinement**			
Up primer occ.	0.90	0.85	0.95
Down primer occ.	0.10	0.15	0.05
A site occupancy	1.00 Mg^{2+}	1.00 Mg^{2+}	1.00 Mg^{2+}
B site occupancy	1.00 Mg^{2+}	0.90 Mg^{2+}	0.90 Mg^{2+}
dGTP occupancy	0.80	0.85	0.80
B-factors			
Me_A/Lig_A[2]	25.22/30.3	23.6/29.0	23.3/27.1
Me_B/Lig_B[2]	20.7/24.3	19.2/23.8	18.9/22.5
Resolution (Å)	1.65	1.66	1.65
No. reflections	54419 (5435)	51387 (4965)	53662 (5308)
R_{work}/R_{free}	0.19/0.22	0.18/0.21	0.19/0.21
Wilson B	26.3	25.8	26.1
Ramachandran			
Favored (%)	97.4	97.7	97.2
Outlier (%)	0.23	0.47	0.23
R.m.s. deviations			
Bond lengths (Å)	0.009	0.009	0.009
Bond angles (˚)	1.2	1.3	1.2

[1]Data in the highest resolution shell is shown in the parenthesis.
[2]B-factor of metal ions and their protein nucleotide ligands.

f A-site titration with Mn^{2+}

	0.06	0.12	0.25	0.5
**PDB Code**	**7U7Y**	**7U7Z**	**7U80**	**7U81**
**Data collection**				
Wavelength (Å)	0.9786	0.9786	0.9786	0.9786
Space group	$P6_1$	$P6_1$	$P6_1$	$P6_1$
Cell dimensions				
a, b, c (Å)	98.61<br>98.61<br>81.80	98.22<br>98.22<br>82.16	98.73<br>98.73<br>82.03	98.31<br>98.31<br>82.09
α, β, γ (˚)	90, 90, 120	90, 90, 120	90, 90, 120	90, 90, 120
Resolution (Å)[1]	42.7 - 1.78 (1.84 - 1.78)	37.8 - 1.67 (1.73 - 1.67)	42.3 - 1.83 (1.90 - 1.83)	42.6 - 1.6 (1.66 - 1.6)
R_{sym} or R_{merge}[1]	0.0781 (1.74)	0.0594 (1.60)	0.1 (1.76)	0.0552 (1.43)
$I/\sigma I$[1]	19.3 (1.4)	23.0 (1.4)	12.9 (1.2)	24.4 (1.4)
$CC^{1/2}$ [1]	1 (0.59)	1 (0.56)	1 (0.59)	1 (0.50)
Completeness (%)	100 (100)	100 (99.9)	99.8 (98.6)	99.8 (99.2)
No. unique reflections[1]	43307 (4269)	52212 (5189)	40035 (3935)	59314 (5852)
**Refinement**				
Up primer occ.	0.75	0.75	0.65	0.65
Down primer occ.	0.25	0.25	0.35	0.35
A site occupancy	0.60 Mn^{2+}	0.65 Mn^{2+}	0.70 Mn^{2+}	0.80 Mn^{2+}
B site occupancy	0.85 Mn^{2+}	0.85 Mn^{2+}	0.85 Mn^{2+}	0.90 Mn^{2+}
dGTP occupancy	0.85	0.85	0.85	0.90
B-factors				
Me_A/Lig_A [2]	31.6/33.6	31.5/33.2	29.5/30.9	22.6/25.7
Me_B/Lig_B [2]	29.2/25.5	27.2/26.3	27.726.0	21.4/22.2
Resolution (Å)	1.78	1.67	1.83	1.60
No. reflections	43305 (4269)	52211 (5189)	40033 (3935)	59306 (5852)
R_{work}/R_{free}	0.18/0.21	0.19/0.22	0.18/0.21	0.19/0.22
Wilson B	29.8	27.0	33.0	24.3
Ramachandran				
Favored (%)	97.9	97.9	98.1	98.1
Outlier (%)	0	0	0.23	0
R.m.s. deviations				
Bond lengths (Å)	0.009	0.01	0.009	0.009
Bond angles (˚)	1.2	1.2	1.2	1.2

[1]Data in the highest resolution shell is shown in the parenthesis.
[2]B-factor of metal ions and their protein nucleotide ligands.

	1	3	6
**PDB Code**	**7U82**	**7U83**	**7U84**
**Data collection**			
Wavelength (Å)	0.9787	0.9787	0.9787
Space group	$P6_1$	$P6_1$	$P6_1$
Cell dimensions			
a, b, c (Å)	98.00	98.27	97.85
	98.00	98.27	97.85
	81.70	81.95	81.55
α, β, γ (˚)	90, 90, 120	90, 90, 120	90, 90, 120
Resolution (Å)[1]	37.7 - 1.55	37.8 - 1.55	37.6 - 1.71
	(1.61 - 1.55)	(1.61 - 1.55)	(1.77 - 1.71)
R_{sym} or R_{merge}[1]	0.0538 (1.2)	0.0546 (1.33)	0.0772 (1.77)
$I/\sigma I$[1]	21.5 (1.4)	22.2 (1.2)	17.0 (1.3)
$CC^{1/2}$ [1]	1 (0.55)	1 (0.49)	1 (0.57)
Completeness (%)	99.8 (97.7)	99.8 (97.9)	100 (99.8)
No. unique	64396	64591	47935
reflections[1]	(6272)	(6331)	(4771)
**Refinement**			
Up primer occ.	0.70	0.65	0.60
Down primer occ.	0.30	0.35	0.40
A site occupancy	0.85 Mn^{2+}	0.95 Mn^{2+}	1.00 Mn^{2+}
B site occupancy	0.90 Mn^{2+}	1.00 Mn^{2+}	1.00 Mn^{2+}
dGTP occupancy	0.90	0.95	1.00
B-factors			
Me_A/Lig_A [2]	19.9/23.9	24.7/25.8	24.6/24.7
Me_B/Lig_B [2]	20.5/21.9	22.7/23.2	23.3/23.2
Resolution (Å)	1.55	1.55	1.71
No. reflections	64394 (6271)	64586 (6331)	47924 (4768)
R_{work}/R_{free}	0.19/0.22	0.19/0.23	0.18/0.24
Wilson B	24.5	25.2	30.3
Ramachandran			
Favored (%)	98.1	98.1	97.9
Outlier (%)	0.23	0.23	0.23
R.m.s. deviations			
Bond lengths (Å)	0.009	0.009	0.009
Bond angles (˚)	1.2	1.2	1.1

[1]Data in the highest resolution shell is shown in the parenthesis.
[2]B-factor of metal ions and their protein nucleotide ligands.
