# Supplementary material for: In crystallo observation of three metal ion promoted DNA polymerase misincorporation
Source: Nat Commun. 2022 Apr 29;13:2346. doi: 10.1038/s41467-022-30005-3 (PMC9054841; doi:10.1038/s41467-022-30005-3)
Supplement: Supplementary file 2 — Description of Additional Supplementary Files [file 41467_2022_30005_MOESM2_ESM.pdf]

Description of Additional Supplementary Files

File Name: Supplementary Movie 1
Description: Cinematic visual of the in crystallo reaction of Pol η misincorporation.

The first 4 seconds were not observed in our study and are theoretical.
